# Using multivariate decoding to go beyond contrastive analyses in consciousness research

**DOI:** 10.3389/fpsyg.2014.01250

**Published:** 2014-10-30

**Authors:** Kristian Sandberg, Lau M. Andersen, Morten Overgaard

**Affiliations:** ^1^Cognitive Neuroscience Research Unit, Hammel Neurorehabilitation Centre and University Research Clinic, Aarhus UniversityAarhus, Denmark; ^2^Institute of Cognitive Neuroscience, University College LondonLondon, UK; ^3^Cognitive Neuroscience Research Unit, Aarhus UniversityAarhus, Denmark; ^4^Center for Cognitive Neuroscience, Department of Communication and Psychology, Aalborg UniversityAalborg, Denmark

**Keywords:** consciousness, multivariate decoding, multivariate pattern analysis, contrastive analyses, MEG, fMRI

## Abstract

Contrasting conditions with and without awareness has been the preferred method for investigating the neural correlates of consciousness (NCC) for decades, yet recently it has been suggested that further insights can be made by moving beyond this method, specifically by meticulously controlling that potential precursors and consequences of the NCC are not mistaken for an NCC. Here, we briefly review the advantages and potential pitfalls of existing paradigms going beyond the contrastive method, and we propose multivariate decoding of neural activity patterns as a supplement to other methods. Specifically, we emphasize the ability of multivariate decoding to detect which patterns of neural activity are consistently predictive of conscious experiences at the single trial level. This is relevant as the “NCC proper” is expected to be consistently predictive whereas processes that are consequences of consciousness may not occur on every trial (making them less predictive) and prerequisites of consciousness may be present on some trials without conscious experience (making them less predictive).

## THE EVOLUTION OF CONTRASTIVE ANALYSIS

In early outlines of contrastive analyses in consciousness research, emphasis was placed on comparing pairs of psychological phenomena of which one was conscious and the other was not (e.g., Baars, 1994). Behavioral characteristics and neural activity could thus be compared between the conscious and unconscious cases. In the case of vision, for instance, neural activity related to masked and unmasked stimulus presentations (Dehaene et al., 2001) or to stimuli presented at various durations (Kjaer et al., 2001) has been investigated. Over the last two decades, methods have evolved so rapidly that it is now difficult to determine what is a natural extension of the contrastive analysis method and what is an alternative method. In this article, we discuss some of the recent developments, and we consider how multivariate decoding, as an extension of or in combination with contrastive analysis, can contribute to identifying neural correlates of consciousness (NCC).

Many recent paradigms were developed in order to avoid confounds present in the original proposals and experiments. For instance, if stimulus duration is varied, the two conditions no longer differ exclusively in terms of the subjective experience of the participant, but also in terms of an important stimulus characteristic, which could be expected to have an impact on conscious as well as unconscious processing (Overgaard, 2004). For this reason, some scientists have preferred paradigms where the physical parameters remain stable, but only the conscious experience varies. This has been done, for instance, using masked stimuli by contrasting trials based on reports of awareness (e.g., Babiloni et al., 2010). Furthermore, in some relatively early studies participants primarily performed objective tasks, and to the extent that awareness reports were used, they were used to confirm that conditions could be treated as subliminal/supraliminal (Dehaene et al., 2001; Kjaer et al., 2001; Silvanto et al., 2005). In contrast, in some later studies, scientists have more often preferred to base analyses on trial-by-trial reports of awareness (or confidence) even when multiple physical stimulus conditions are used (Christensen et al., 2006; Koivisto, Mäntylä et al., 2010). The use of awareness reports can be seen as a necessary consequence of the wish to control for physical parameters. Methodologically speaking, these reports separate conditions when trials no longer differ in terms of objective characteristics. But their use is also partly a consequence of theoretical arguments in favor of the crucial role of awareness ratings as a key measure of validity in consciousness research (Overgaard, 2006, 2010). Some scientists even prefer to keep accuracy stable so that *only* the level of awareness varies between conditions (Lau and Passingham, 2006; Lau, 2008) or to examine the correlates of accuracy and awareness separately while ensuring that mask and stimulus have very different neural signatures (Hesselmann et al., 2011).

Common to most recent studies is that the need to control for potential confounds has resulted in a shift from the examination of complete unawareness versus complete awareness to the examination of smaller differences in graded awareness ratings or changes in the probability of obtaining reports of awareness. As the change between conscious and unconscious perception occurs more suddenly across stimulus intensity for the attentional blink (than for masking), this paradigm has sometimes been preferred (e.g., Sergent et al., 2005) although others are reluctant to use the paradigm as they suspect it reflects failure to attend (possibly conscious) perception (e.g., Lamme, 2006). Bistable perception provides another method for ensuring both conscious and unconscious perception under equal stimulation conditions. Many earlier studies using ambiguous perception examined differences in neural activity related to ambiguity/non-ambiguity (Lumer et al., 1998) or reversals of perception (Kornmeier and Bach, 2004), but some have also compared neural activity related to one perceptual state versus another (Andrews et al., 2002; Sterzer and Rees, 2008; Sandberg et al., 2013).

## RECENT DEVELOPMENTS

Recently, it has been argued that it is possible that studies using contrastive analyses cannot distinguish between a NCC and its prerequisites (NCC-pr) and consequences (NCC-co; Aru et al., 2012). An NCC-pr is neural activity associated with task specific initial processing (which predicts later conscious experiences) whereas an NCC-co is neural activity related to a process that occurs for conscious stimuli only, for instance encoding in working memory. Aru et al. (2012) have argued that by manipulating stimulus processing in various ways, NCC-pr and NCC-co should change, but the NCC should remain stable. In one experiment, Melloni et al. (2011) manipulated the stimulus expectation across conditions and found that an early EEG component (around 100 ms) only reflected differences between seen and unseen stimuli when there was no expectation of the stimulus, and similarly a later component (the P300) only correlated with awareness when stimuli had to be encoded in working memory, but not when a representation was already present. In contrast, a component between the two, at around 200–300 ms, correlated with conscious perception independently of condition. This indicated that the first component was an NCC-pr, the middle component at 200–300 ms a likely NCC candidate, and the P300 an NCC-co.

Although this method for moving beyond contrastive analysis is certainly novel and useful, it assumes one can evoke the same experience by means of multiple, very different manipulations. However, there is no guarantee that the experience is identical even if the same proportion of awareness responses is obtained across conditions. Ratings of awareness can be viewed as a decision process in which evidence is gathered for a particular response (e.g., Lau, 2008), for example “seen,” but when different manipulations are made, the decision axis is no longer shared, and thus it is unknown if the NCC can be expected to remain unchanged (Jannati and Di Lollo, 2012). A potential solution to this could be the use of more detailed awareness ratings, but it may also be possible to improve the paradigm in general using decoding approaches as we will return to later.

Accordingly, we still have no paradigm to investigate NCCs without potential systematic confounds. Newer paradigms, to some degree, have solved problems in previous paradigms, yet have introduced new ones. For this reason, we argue that converging evidence across multiple paradigms is essential in the search for the “NCC proper” (Overgaard, 2011).

## MULTIVARIATE DECODING

Here, we use the term multivariate decoding [also sometimes referred to as multivariate/multi-voxel pattern analysis, pattern classification, “brain reading,” or simply decoding (Haynes and Rees, 2006; Norman et al., 2006; Haynes, 2009)] as an umbrella term for a group of analysis techniques for which the goal, in this context, is to decode the conscious experience of a participant based on large amounts of brain data. We will exemplify the general logic behind multivariate decoding by example of a within-subject decoding.

**FIGURE 1:**
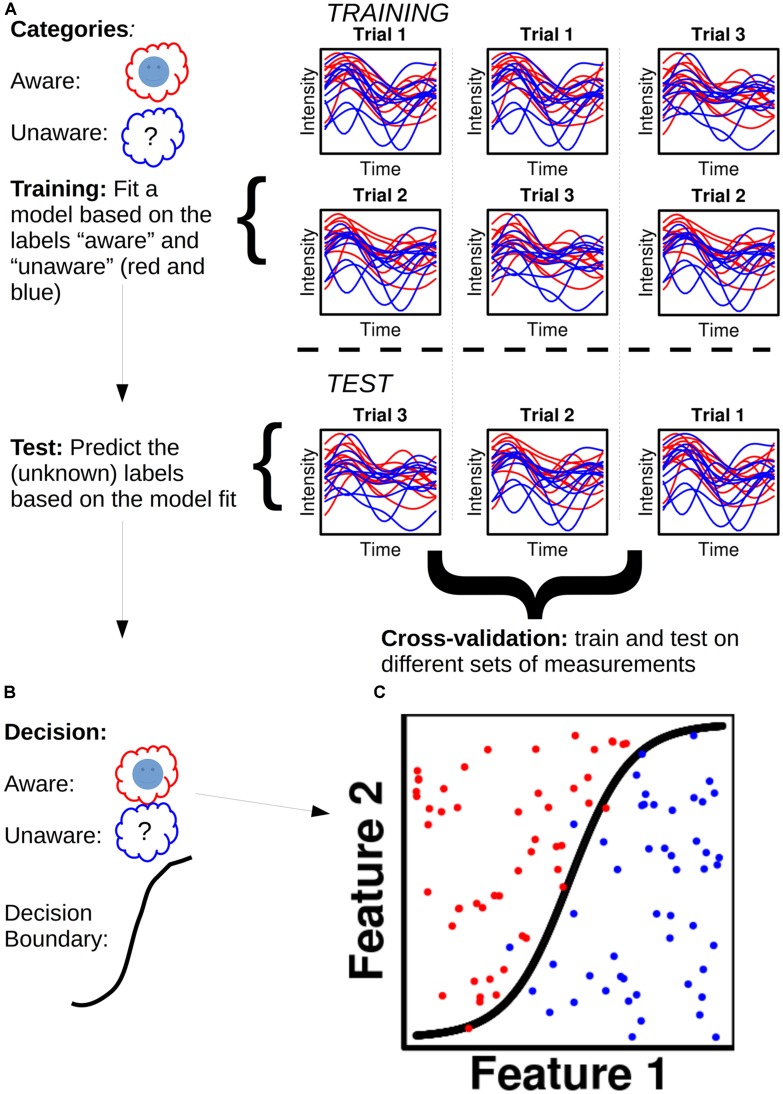
**Illustration of a hypothetical classification analysis and the steps involved. (A)** The classifier is to distinguish between two categories: “Aware” (red) and “Unaware” (blue). Trials are separated into training and test sets in three different ways to ensure cross-validation. The plots show hypothetical activity developing over time courses for ten trials of aware and unaware respectively. **(B)** The decisions reached based on the model fit in the training set. **(C)** Classification is performed for 100 trials (50 aware and 50 unaware) with a non-linear decision boundary.

Take an MEG dataset (**Figure 1**), for instance, of a subject with *x* epochs of class **A** (e.g., “aware”) and *x* epochs of class **B** (e.g., “no awareness”): each data point of each epoch is called a feature. For a given dataset with *n* sensors/sources and *t* time points, one will thus have *n* X *t* features for each epoch. The dataset is then divided into two parts, a training set (often 90% of the data) and a test set (the remaining 10%; **Figure 1A**). A model is fitted to the training set and each feature is assigned a weight. Dependent on the sign of a given weight, it raises the posterior probability of a given epoch to belong to class **A** or **B**, respectively. The fitted training set, with its feature weights, is then used to predict the class of each epoch for the test set (**Figure 1B**). The predicted class label for a given epoch is the class label that has the highest posterior probability assigned to it when the feature weights for that epoch are summed together. One can then obtain a classification score, which is the percentage of correctly classified epochs. **Figure 1C** shows an example of this. To test the generality of the classification score, one can cross-validate the score by dividing the data set into training and test sets in different ways.

We believe that multivariate decoding has a role in neuroscientific consciousness research for several reasons and in the following we will go through these. We will, however, first emphasize that decoding results should be interpreted with care: although a given mental state can be decoded above chance from particular neural activity, this does not in itself imply a causal relationship. In this sense, multivariate decoding shares some of the limitations of correlation studies. Multivariate decoding, nevertheless, opens up new possibilities that have not previously been available.

## INCREASED SENSITIVITY OF MULTIVARIATE DECODING

One main advantage of multivariate decoding is the greater sensitivity than that of traditional mass-univariate approaches typically used in contrastive analyses (i.e., the testing of single variables one at a time; Haynes and Rees, 2006; Norman et al., 2006). Multivariate decoding is more sensitive that univariate testing due to pooling of information and the informativeness of the co-variance of the features (Haynes and Rees, 2006). Furthermore, univariate tests typically test for linear relationships whereas the nature of the relationship does not need to be specified to achieve successful decoding (Haynes, 2009). The advantage of multivariate decoding in consciousness research has been shown for fMRI where Haynes and Rees (2005) showed that decoding based on V1–V3 voxels combined was more predictive of perception during binocular rivalry than decoding based on the combined mean of the same voxels. Similarly, using MEG Sandberg et al. (2013) showed that perception during binocular rivalry can be decoded at an accuracy just a few percent below peak decoding accuracy (around 75%) using just 10 occipital sensors, which were individually at chance (below 51.5%).

At its core, all univariate testing regards data points as independent of one another, which is evidently false for both MEEG and fMRI data. It is precisely the heavy spatial and temporal correlations of neuroimaging data that make them fit for multivariate analyses. In contrast to univariate tests, multivariate tests can facilitate the information contained in the temporal and spatial dependencies between data points in both sensor and source space (MEEG) and in voxel space (fMRI) in a single test.

## FINDING CONSISTENT CORRELATES USING MULTIVARIATE DECODING

Multivariate tests are more sensitive to differences between conditions that are present during *all* epochs, and that they are less sensitive to differences between conditions that are only present during *some* of the epochs. Indeed, Haynes (2009) emphasized that a core NCC (or “NCC proper”) should in principle be able to predict a conscious state perfectly. From this it follows that higher decoding accuracy is generally a sign of greater representational accuracy although it must be emphasized that care should be taken when comparing decoding accuracies across different brain areas, and there are several aspects to consider. For instance, Kamitani and Tong (2006) found that perceived motion direction was only decoded as well from MT+ as from earlier visual areas V1–V4 when the same number of voxels was used. Indeed, a later article by Smith et al. (2011) mention that when comparing fMRI decoding accuracies across conditions, participants, or brain regions, it is important that several factors are controlled for including the number of voxels and stimulus repetitions (and we might add that not only the number of spatial, but also the number of temporal, features should be controlled for). Additionally, they specifically emphasize the importance of controlling for or taking into account the mean amplitude of the component of interest as they show that decoding accuracy increases as a function of mean amplitude even if specificity is not increased. The function with which classifier accuracy increases as a function of response amplitude (measured as percent signal change for fMRI) can nevertheless be estimated and compared across areas for a more valid comparison of decoding accuracy. A simpler, but not always feasible solution is to compare components of equal amplitude.

A note of caution is necessary, however: even when mean amplitude is controlled for, the obtainable signal from two components may differ in their signal-to-noise ratios (for instance, if the angle of the neurons prevents a good signal in MEEG). This necessitates that one is cautious when interpreting differences in accuracy between MEEG components unless one has a good way to estimate differences in noise ceilings. Such estimations are possible with encoding models (Kay et al., 2008) or with representational similarity analysis (Nili et al., 2014), but it is presently an unresolved issue for decoding models and further work in this field is important for ensuring the validity of comparisons of decoding accuracies. It should be emphasized that the issue is not likely to be dramatic and presently a rough estimate of noise ceiling may be achieved by prior knowledge of decoding accuracies across different tasks for various brain regions/components.

**FIGURE 2:**
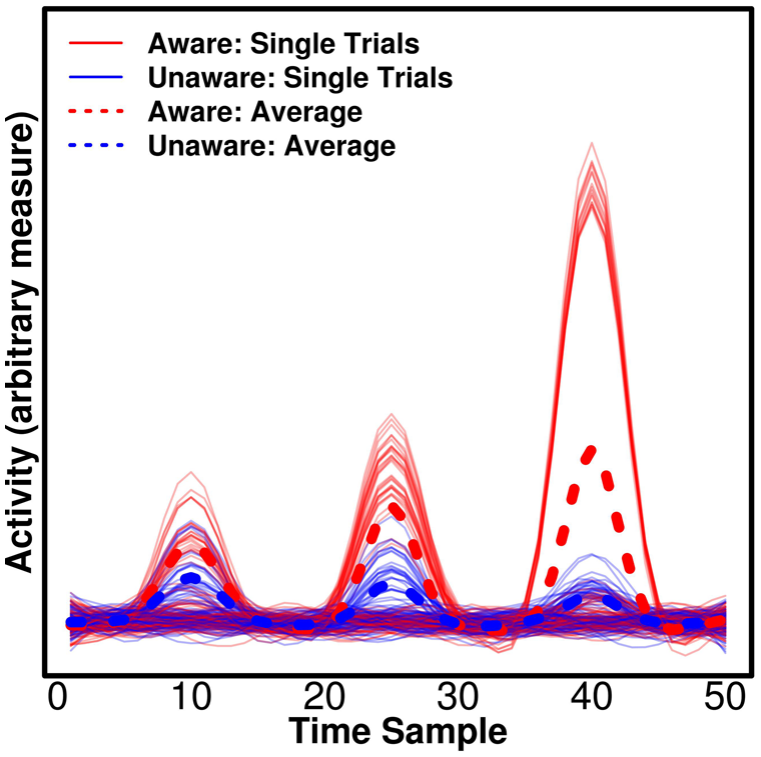
**Consistency of the neural correlates of consciousness (NCC).** Three simulated, hypothetical signals of differing consistency and strength are plotted. All could be candidate NCC, thus reflecting differences between trials classified as “aware” and “unaware” by a participant. For the first component, there is a small average difference, but the component is not consistently larger for “aware” trials, making it unlikely that the component reflects awareness. The component could reflect a prerequisite for consciousness (NCC-pr) as it has to be present for awareness, but it does not guarantee awareness. For the second component, there is a medium average difference, and the component is consistently larger for “aware” trials. On the single trial level, the component thus reflects awareness and it may thus be an actual NCC. Finally, for the third component, there is a large average difference, but the component is only found on a subset of “aware” trials, and it does thus not consistently reflect awareness. The component could thus reflect processes that are consequences of awareness (NCC-co), which occur exclusively for “aware” trials, but may not occur on every single aware trial. Note that traditional univariate statistics based averaged participant-specific averages would erroneously find more evidence for the last component being the NCC proper in this example.

Univariate tests are of course sensitive to differences that are present on all epochs, but crucially they can, in addition, be sensitive to differences that appear only on some epochs, but show some average difference between conditions (e.g., aware/unaware). This has important implications for the attempt to separate NCC-pr, NCC, and NCC-co. In **Figure 2**, we show simulated data with three components for which there are average differences between trials reported as “aware” and “unaware” by a participant. We would expect the actual NCC to vary consistently with the conscious experience – whenever the participant has an experience of the stimulus, the relevant component should reflect this. The NCC-pr, however, might be present without the NCC on some trials (i.e., one particular prerequisite of conscious experience was present on a trial, but perhaps some others were not, and the participant thus had no experience) in which case the component becomes an unreliable predictor and should not be assigned high weights by the classifier when all data are taken into account, and it should produce suboptimal decoding accuracy when used to train/test the classifier alone. This corresponds to the first component in **Figure 2**. The NCC-co, on the other hand, might not occur after each single NCC component (even if it occurs after some NCC components), and it should never occur without an NCC component. It is thus expected to be similarly suboptimal for decoding even if it produces very large responses on some trials and a large average difference. This corresponds to the third component in **Figure 2**. The actual NCC is thus expected to be consistently the most predictive at the single trial level even if it does not produce the largest average difference. This corresponds to the second component in **Figure 2**. As mentioned above, multivariate decoding approaches are able to identify the most consistent correlates, but traditional univariate analyses typically base statistics on participant-specific means and would in our example find significant evidence in favor of the third component even though it only occurs on some trials. Importantly, if the aim is to compare components, as in our example (**Figure 2**), univariate tests are not readily interpretable. There is no straightforward interpretation of what a difference in amplitude between components means (Luck, 2014). In comparison, the interpretation of differences in decoding accuracy is straightforward – it simply means that the pattern holds more information about the label of the state, say “aware” or “unaware.”

In cases where the confounding processes occur on every single trial with an awareness response, multivariate decoding on its own will not be able to distinguish between NCC and NCC-pr/NCC-co as all responses could be equally predictive. For this reason, we believe that the optimal paradigm is a combination of decoding and the methods suggested by Melloni et al. (2011) and Aru et al. (2012). One way to combine methods would be to use cross-task decoding – i.e., using several tasks resulting in similar conscious experiences and training/testing on different tasks using a leave-one-out procedure. In this case, decoding performance should be best for components that generalize across experimental contexts.

Using multivariate decoding on MEG data, a study by our group have found that conscious experience during binocular rivalry was predicted relatively accurately by activity around 130–320 ms after stimulus onset and that an earlier and a later component was not consistently predictive (Sandberg et al., 2013). In an additional (ongoing) MEG study, multivariate decoding furthermore showed that activity around this time was the most predictive of small, graded differences in the clarity of conscious experience on the single trial level (Andersen et al., in preparation). Similarly, decoding can be used on different brain areas in turn in order to compare how consistently predictive these are separately (and/or combined; Norman et al., 2006). For binocular rivalry, this was done for V1–V3 by Haynes and Rees (2005) and across the cortex by Sandberg et al. (2013). Lastly, it should be acknowledged that when doing multivariate analyses, “decoding” is not strictly necessary. There are ways of doing “encoding” as well, where one can extract parameters from the model, as in classical univariate models. Encoding applications are at the moment, however, less available than decoding applications, both theoretically and practically, but see Allefeld and Haynes (2014) for a novel approach.

## OTHER POSSIBILITIES USING MULTIVARIATE DECODING

The use of multivariate decoding opens up for potential research, which would otherwise be difficult or even impossible to conduct. For MEG, conscious experience can be decoded using only a few milliseconds of data gathered within the first 200 ms after stimulus presentations (Sandberg et al., 2013, 2014). Particularly, if near-perfect, near real-time decoding can be achieved, it may be possible to exploit such speed in the control of brain-computer interfaces. At present, one study was able to achieve above 85% decoding accuracy for three of eight participants (and around 95% for one; Sandberg et al., 2013). In comparison, univariate decoding (i.e., using the single best sensor at the single best time point) resulted in lower accuracies (around 10% lower), and would furthermore require both time point and sensor to be specified in advance. Additionally, other studies have shown cases in which multivariate decoding is above chance in the absence of an average activity difference (Sterzer et al., 2008).

Because decoding can be accomplished prior to report, it raises the possibility that an MEG based brain-computer interface could be used to generate changes in the environment even before they are produced by the motor behavior of the individual, which could be of key importance in the study of overt behavior and sense of agency. Furthermore, neural correlates can be analyzed before and after the preparation to report in the attempt to filter out correlates of introspection, metacognition, and motor preparation. And finally, fast and accurate decoding allows for manipulations of stimuli or brain activity (using TMS, for instance) around the time where an event is experienced, but before it is reported, and it may allow for the study of awareness without report.

Haynes and Rees (2006) emphasized the importance of the then unresolved issue of how well activity generalizes over time, across situations (paradigms) and even across participants. This can be examined by conventional methods using correlations, but decoding provides a method of examining whether minor changes are critical or whether the overall patterns are generally maintained. Haynes and Rees (2005) used fMRI to examine drops in decoding accuracy across days, but the first long-term study was conducted by Sandberg et al. (2014), who found that the decrease in decoding accuracy within participants across 2.5 years was only around 1%, which was comparable to the drop across a few days. This study also found that the drop when attempting to generalize across participants (even at the source level) was much greater (around 10%). Further studies examining whether minor details in patterns of activity predict related changes in perceptual experience can be used to address theoretical questions about multiple realization in the brain.

It has also been established that it is possible to decode the conscious experience of one individual using a classifier trained on a different individual although the accuracy is lower than for within-individual decoding (Poldrack et al., 2009; Haxby et al., 2011; Sandberg et al., 2013, 2014). This opens up possibilities that so far have been outside the reach of cognitive neuroscience methods. One might apply multivariate decoding to investigate whether neural correlates generated in experiments using one paradigm can be used to train a classifier to decode the experience in other paradigms as we discuss above. Furthermore, between-participant decoding opens possibilities of decoding across groups for which it is uncertain whether one has conscious experiences, such as vegetative or minimally conscious patients. When consciousness has been examined in non-human animals, methods such as flash suppression have been used to ensure the validity of report as the stimuli are bistable but conscious perception can be manipulated by the experimental setup (Sheinberg and Logothetis, 1997). Such or similar methods could in principle also be used with patients, and it could be possible to decode both within individuals but also to examine how well classifiers generalize from healthy individuals to reduced consciousness patients. Here again, the improved accuracy of multivariate decoding provides an advantage compared to univariate approaches.

## Conflict of Interest Statement

The authors declare that the research was conducted in the absence of any commercial or financial relationships that could be construed as a potential conflict of interest.
